# In Vitro Studies Regarding the Effect of Cellulose Acetate-Based Composite Coatings on the Functional Properties of the Biodegradable Mg3Nd Alloys

**DOI:** 10.3390/biomimetics8070526

**Published:** 2023-11-04

**Authors:** Alexandru Streza, Aurora Antoniac, Veronica Manescu (Paltanea), Robert Ciocoiu, Cosmin-Mihai Cotrut, Marian Miculescu, Florin Miculescu, Iulian Antoniac, Marco Fosca, Julietta V. Rau, Horatiu Dura

**Affiliations:** 1 Faculty of Material Science and Engineering, National University of Science and Technology Politehnica Bucharest, 313 Splaiul Independentei Street, District 6, 060042 Bucharest, Romania; alexandru_streza@yahoo.com (A.S.); veronica.paltanea@upb.ro (V.M.); ciocoiurobert@gmail.com (R.C.); cosmin.cotrut@upb.ro (C.-M.C.); marian.miculescu@upb.ro (M.M.); f_miculescu@yahoo.com (F.M.); antoniac.iulian@gmail.com (I.A.); 2 Faculty of Electrical Engineering, National University of Science and Technology Politehnica Bucharest, 313 Splaiul Independentei Street, District 6, 060042 Bucharest, Romania; 3 Academy of Romanian Scientists, 54 Splaiul Independentei Street, District 5, 050094 Bucharest, Romania; 4 Istituto di Struttura della Materia, Consiglio Nazionale delle Ricerche (ISM-CNR), Via del Fosso del Cavaliere 100, 00133 Rome, Italy; marco.fosca@ism.cnr.it; 5 Department of Analytical, Physical and Colloid Chemistry, Institute of Pharmacy, I.M. Sechenov First Moscow State Medical University, Trubetskaya Street 8, Build. 2, 119048 Moscow, Russia; 6 Faculty of Medicine, Lucian Blaga University of Sibiu, 2A Lucian Blaga Street, 550169 Sibiu, Romania; horatiudura@yahoo.com

**Keywords:** magnesium alloys, biodegradable alloys, Mg3Nd alloys, cellulose acetate composite coatings, corrosion, surface

## Abstract

Magnesium (Mg) alloys are adequate materials for orthopedic and maxilo-facial implants due to their biocompatibility, good mechanical properties closely related to the hard tissues, and processability. Their main drawbacks are the high-speed corrosion process and hydrogen release. In order to improve corrosion and mechanical properties, the Mg matrix can be strengthened through alloying elements with high temperature-dependent solubility materials. Rare earth elements (RE) contribute to mechanical properties and degradation improvement. Another possibility to reduce the corrosion rate of Mg-based alloys was demonstrated to be the different types of coatings (bioceramics, polymers, and composites) applied on their surface. The present investigation is related to the coating of two Mg-based alloys from the system Mg3Nd (Mg-Nd-Y-Zr-Zn) with polymeric-based composite coatings made from cellulose acetate (CA) combined with two fillers, respectively hydroxyapatite (HAp) and Mg particles. The main functions of the coatings are to reduce the biodegradation rate and to modify the surface properties in order to increase osteointegration. Firstly, the microstructural features of the experimental Mg3Nd alloys were revealed by optical microscopy and scanning electron microscopy (SEM) coupled with energy-dispersive spectroscopy. Apart from the surface morphology revealed by SEM, the roughness and wettability of all experimental samples were evaluated. The corrosion behavior of the uncoated and coated samples of both Mg3Nd alloys was investigated by immersion testing and electrochemical testing using Simulated Body Fluid as the medium. The complex in vitro research performed highlights that the composite coating based on CA with HAp particles exhibited the best protective effect for both Mg3Nd alloys.

## 1. Introduction

Recently, magnesium (Mg) alloys have been considered the most adequate materials in the biomedical field because they exhibit high specific strength, are lightweight, and have good castability, heat dissipation, and machinability [1,2,3]. It was shown that the magnesium ion release process has a beneficial effect on angiogenesis and osteogenesis, a fact that makes this material adequate for better orthopedic fixation [4,5,6,7]. Their main drawback is considered to be the high-speed degradation process, which occurs when the Mg implant is introduced in physiological media, followed by hydrogen evolution [8]. This last-mentioned effect should be carefully controlled by considering its potential to induce necrosis of healthy tissue. Magnesium cannot be combined during implant manufacture with other parts made of such materials as copper (Cu), titanium (Ti), aluminum (Al) alloys, and stainless steel due to galvanic corrosion. It was noticed that high-purity magnesium exhibits the lowest corrosion rate because it contains α-Mg in the microstructure [9]. The material properties can be controlled and modified in a desired fashion by adding alloying elements, whose selection must be carefully performed by considering the difficulty of Mg alloy manufacturing due to its increased pyrophoricity when it is used as powder.

It is well known that Mg chemically reacts with alloying compounds and generates intermetallic phases with an important influence on the material microstructure. In some cases, the mechanical properties of the alloys can be improved due to precipitation hardening and solid-solution or grain-refinement strengthening phenomena [10]. The Mg matrix can be strengthened through alloying with high temperature-dependent solubility materials [11]. The element’s atomic size with respect to Mg and its valence greatly influence the overall solubility. By modifying the alloying element number and weight, alloys with different properties and corrosion behavior can be obtained. An important alloying element with good biocompatibility is Zinc (Zn). The tolerance limit for this element in Mg was established at 2.5 wt.% [12]. The literature evidenced that the corrosion resistance decreased when the Zn content was increased from 1 to 3 wt.% [13]. If it is higher than 3 wt.%, stress corrosion cracks occur. The Zn percent must be carefully checked because it acts in the biocorrosion process by decreasing the hydrogen evolution. Zn is highly biocompatible, being present in the human body in bones and muscles. Due to its higher Young modulus compared to that of Mg, a non-uniform load transfer between the implant and bone tissue was observed [14]. Zirconium (Zr) is used for grain refinement, although the literature shows that it promotes anode chemical reactions and can damage the surface coatings. Its quantity must be tuned in order to have reduced corrosion rates. 

Rare earths (RE) contribute to mechanical property improvement, altering the material texture and influencing the degradation behavior. In [15,16], it was observed that RE addition facilitates the enhanced performance of Mg alloys when they work at high temperatures. The most used REs as alloying elements in Mg-based alloys are cerium (Ce), neodymium (Nd), yttrium (Y), gadolinium (Gd), lanthanum (La), and praseodymium (Pr). RE forms a stable ternary eutectic compound Mg-Zn-RE, which improves the creep resistance and castability of Mg-Zn-Zr alloy [17]. Loos et al. [18] investigated the Y biocompatibility based on in vitro and in vivo tests on Mg stents. They found that accumulation in the gallbladder and liver is possible if a high Y concentration is used. Also, Y caused increased eosinocyte levels in the blood and has a detrimental effect on implant biocompatibility. Other studies showed that the amount of added Nd must be controlled because the formation of the Mg_3_Nd phase in the binary alloy Mg-Nd increases its corrosion rate. As an overall finding, its corrosion rate is much more reduced in comparison with that obtained through La or Ce addition [1]. Nd determines the appearance of a shielded oxide layer at the surface of Mg alloys. Another RE with a positive impact on corrosion resistance is Gd because, by its addition, a high solid solution in Mg occurs. It was noticed that Gd provides good corrosion resistance only if its content is lower than 10 wt.% [19]. Due to its toxicity in the case of medical implants, it can be added only in an amount lower than 1 wt.% [20]. Cerium decreased the corrosion rate in the case of Mg-Zn-Zr and Mg-Al-Zn alloys by reducing the grain size [21]. Unfortunately, in vivo studies performed on murine animal models show evidence that Ce disturbed the function of the liver, brain, and kidneys. It is well known that an increase in the metallothioneins (MTs) induced by heavy metal exposure can offer protection against oxidative stress. At the same time, glutathione (GSH) is another antioxidant that plays an important role in cellular defense. In [22], an increase in GSH and MT concentration was evidenced for cerium-treated ICR mice. The authors concluded that Ce could be considered oxidant toxic for mice because it generated hepatic MT synthesis, decreased hepatic Lipoperoxide concentration, and increased hepatic GSH concentration, among others. Willbold et al. [23] investigated the biocompatibility of the following binary alloys: Mg-La, Mg-Nd, and Mg-Ce. They observed that these alloys showed no toxicity against the healthy tissue, although the Mg-Ce exhibited the most reduced cell viability. In conclusion, the authors stated that RE addition improves the corrosion rate and mechanical properties, but their content should be carefully tuned in order to avoid unwanted biological effects. He et al. [24] investigated the effect of Zn addition on the mechanical properties and microstructure of the Mg-3Y-2Nd-0.5Zr alloy. They added 0.5 wt.% Zn and applied a solid solution treatment at 525 °C and an aging step at 200 °C. Zn-Zr precipitates with a rod-like and rectangular block-like shape were noticed in the α-Mg matrix, and many needle-like β phases were present at the terminal parts of Zn-Zr precipitates in the case of samples, for which an aging treatment was applied. Chen et al. [25] modified the Y concentration (0, 0.03, 0.06, 0.12 at.%) in the Mg-Nd-Zn-Zr alloy. A phase consisting of Mg_24_Y_5_ appeared in the case of Y-containing alloys. Supplementary, a grain-refining process was put in evidence. The UTS value increased directly with the Y content from 300 MPa (0 at.%) to 320 MPa (0.12 at.%). 

The insight gained herein may help equip practitioners with an understanding of the structure–property relationship, towards rational material selection and manipulation [26].

Kirkland et al. [27] conducted a survey on the bio-corrosion rates of magnesium alloys. They analyzed the dissolution rates and mass loss of different Mg alloys using Minimum Essential Medium (MEM) incubated at 37 °C in 5.0% CO_2_. In the case of the Mg1Nd alloy, all the mass loss values measured at 3, 7, and 14 days were below 1 mg/cm^2^/day. Other Mg-RE alloys exhibited an increased mass loss, such as Mg1Ce with a mass loss of about 1 mg/cm^2^/day and Mg0.5Y with a value of 2 mg/cm^2^/day. The highest mass loss was obtained for the Mg5Ca alloy with a value higher than 10 mg/cm^2^/day, measured after 3 days of immersion. It can be concluded that RE addition has a beneficial effect on increasing corrosion resistance.

The degradation rate of Mg-based alloys can be reduced through coating deposition. Different coatings, such as bioceramic, polymeric, and polymeric-based composites, are reported in the literature as having a positive effect on different magnesium-based alloy biodegradations [28,29,30,31,32,33,34,35]. Polymeric coatings can be of natural or synthetic origins and can be used to deliver different drugs or treatments. Some of the most common synthetic polymers are cellulose acetate (CA), polylactic acid (PLA), polycaprolactone (PCL), and poly(lactide-co-glycolic) acid (PLGA), and they can be combined with calcium phosphate (Ca-P) or Mg powders in order to improve the corrosion and wear resistance of the alloy [31]. Cellulose acetate is a biocompatible, non-toxic polymer that forms transparent films. Membranes manufactured from this polymer are characterized by a hydrophilic nature, chemical stability, good mechanical properties, reduced protein adsorption, and protein transport capabilities. Neacsu et al. [32] applied a CA coating on the Mg-Ca-Mn-Zr alloy through a dipping process. The potentiodynamic polarization investigation proved that this coating increased the corrosion resistance of the alloy. Supplementary, good cytocompatibility on the MC3T3-E1 cell line was noticed. It was concluded that even though only a few studies were reported in the literature, CA exhibits high biocompatibility and can be used in safe conditions as a coating for scaffolds or implants. Various groups have proposed other synthetic polymers to be used as coatings for degradable Mg alloys, as presented in Table 1.

**Figure 1:**
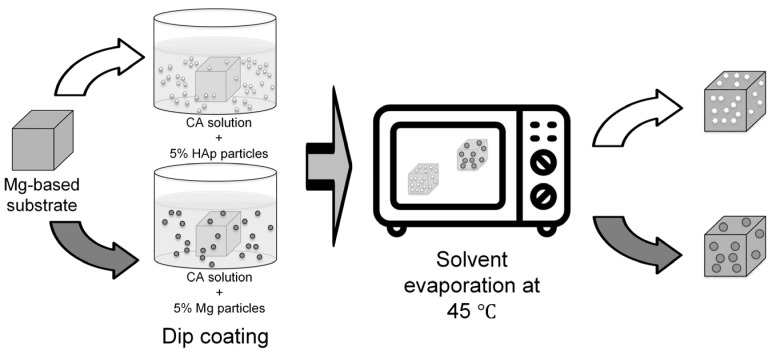
Schematic diagram of the preparation method for the coated Mg-based samples.

The present investigation consists of polymeric-based composite coatings made of CA combined with two bioactive fillers, such as HAp or Mg particles, applied on the surface of two Mg3Nd alloys to reduce the biodegradation rate and to increase the surface osteointegration (Figure 1). 

## 2. Materials and Methods

In this study, two experimental magnesium alloys from the system Mg3Nd, with Y, Zr, and Zn as other alloying elements (Mg-Nd-Y-Zr-Zn), with elemental compositions given in Table 2, were selected to be characterized and tested to evaluate their potential as raw materials for trauma implant fabrication. The alloys were obtained by sand/gravity casting, as described previously [38,39].

To reduce the rapid degradation in physiological environments with a pH between 7.4 and 7.6, the investigated alloys were coated using a CA solution with HAp (Sigma-Aldrich, St. Louis, MO, USA) or Mg (STREM CHEMICALS Inc., USA) particles. The polymer solution (at a concentration of 12 wt.%) was obtained under vigorous mechanical stirring by dissolving CA (Sigma-Aldrich, St. Louis, MO, USA—30% acetylation degree) in N,N′-dimethylformamide (Merck, Darmstadt, Germany). The Hap and Mg particles were dispersed in the polymer solution in a proportion of 5 wt.% related to the amount of cellulose acetate. For the Mg3Nd alloy coating, samples with dimensions of 15 × 15 × 5 mm^3^ were immersed in the obtained polymer solution, followed by evaporation of the solvent for 3 days at a temperature of 45 °C. The operation was repeated three times to obtain a homogeneous coating. After the coating process, the samples were coded as follows: *Mg3Nd_A_CAHAp*–Mg3Nd_A alloy with a composite coating based on CA reinforced with HAp, *Mg3Nd_A_CAMg*–Mg3Nd_A alloy with a composite coating based on CA reinforced with Mg, *Mg3Nd_B_CAHAp*–Mg3Nd_B alloy with a composite coating based on CA reinforced with HAp, and *Mg3Nd_B_CAMg*–Mg3Nd_B alloy with a composite coating based on CA reinforced with Mg. Before characterization and testing, the uncoated and coated alloys were washed with ethanol (Sigma-Aldrich, St. Louis, MO, USA).

### 2.1. Materials Characterization

The Mg3Nd alloys’ microstructure was evaluated using an Olympus BX51 optical microscope (Olympus Life and Materials Science Europa GMBH, Hamburg, Germany) and an FEI QUANTA INSPECT F microscope (FEI Company, Eindhoven, The Netherlands) equipped with an EDS analyzer. For microstructural analysis, the metallographically prepared magnesium alloy samples were chemically etched with a solution of 2.4 g of picric acid, 18 mL of glacial acetic acid, 76 mL of ethanol, and 18 mL of distilled water. Also, the surface morphology of the coated Mg3Nd alloys and the layer thickness were highlighted using scanning electron microscopy (SEM).

### 2.2. Corrosion Behavior of the Experimental Samples

#### 2.2.1. Immersion Test

To evaluate the mass loss, we used five cuboid samples from each type of uncoated and coated alloy with dimensions of 15 × 15 × 5 mm^3^ (length × width × thickness). The test was carried out at 37 ± 1 °C for 72, 168, and 336 h, each sample being introduced into 50 mL of test medium. We used Kokubo’s simulated body fluid solution (SBF, prepared in our laboratory according to [40]) as a test medium. The solution was changed daily to simulate the human body’s environment as accurately as possible.

The mass loss was calculated using the following equation:

(1) $$ML=\frac{m_{0}-m_{f}}{m_{0}}\times 100$$

where *ML*—the mass loss (%), *m*_0_—initial mass value, and *m_f_*—final mass value.

#### 2.2.2. Electrochemical Tests

The corrosion behavior of the two Mg3Nd alloys was investigated with a PARSTAT 4000 Potentiostat/Galvanostat device (Princeton Applied Research, Oak Ridge, TN, USA) in SBF solution (pH of 7.4), following the ASTM G5-94 (2011) standard at 37 ± 0.5 °C in a CW-05G (Jeio Tech) recirculation and heating bath. A three-electrode cell comprised of a counter electrode consisting of a platinum foil, a saturated calomel electrode, SCE, and the sample as a working electrode was involved. 

The corrosion potential (*E_corr_*) and corrosion current density (*i_corr_*) were extrapolated from the Tafel curves that were experimentally measured in the condition of an applied potential range of ±250 mV vs. EOC (potential rich at quasi-equilibrium) at a speed of 1 mV/s. The EOC was measured for one hour until the system reached a quasi-equilibrium state. The polarization resistance *R_p_* (kΩcm^2^) was determined as indicated in ASTM G59-97 according to Equation (2):

(2) $$R_{p}=\frac{1}{2.3}\frac{\beta_{a}\beta c}{\beta_{a}+\beta c}\frac{1}{i_{corr}}$$

*β_a_* (mV)—the slope of the Tafel anodic curve, *β_c_* (mV)—the slope of the Tafel cathodic curve.

The corrosion rate was computed as presented in Equation (3) and ASTM G102-89 for uncoated samples:

(3) $$CR=K_{i}\frac{i_{corr}}{\rho }EW$$

*CR*—the corrosion rate (mm/year), *K_i_* = 3.27 × 10^−3^ (C^−1^), *i_corr_*—the corrosion current density (μA/cm^2^), *ρ*—the material density (g/cm^3^), and *EW*—the equivalent weight (g). 

Another important parameter, which can be determined only in the case of coated samples, is the protective efficiency (Equation (4)):

(4) $$P_{e}=\left(1-\frac{i_{corr,coating}}{i_{corr,substrate}}\right)100$$

*i_corr,coating_* (μA/cm^2^)—the corrosion current density for the Mg3Nd alloy coatings and *i_corr,substrate_* (μA/cm^2^)—the corrosion current density for the Mg3Nd alloys (substrate).

### 2.3. Surface Properties of the Experimental Samples

The equipment involved in the material *wettability investigations* was the Krüss Drop Shape Analyzer-DSA100 (A. Krüss Optronic GmbH, Hamburg, Germany). This device is suitable for experiments with three wetting agents: water (W), diiodomethane (DIM), and ethylene glycol (EG). All the measurements were performed in laboratory conditions (23 ± 5 °C, humidity of 45 ± 5%). We analyzed six samples for both Mg3Nd alloys (two control samples, two samples coated with composite coatings based on CA and 5% HAp particles, and two samples coated with composite coatings based on CA and 5% Mg particles). For each wetting agent, 12 successive measurements were performed, and the obtained images were analyzed based on ImageJ 1.50 software (National Institutes of Health, Bethesda, MD, USA). The Owens, Wendt, Rabel, and Kaelbe (OWKR) procedure was applied to compute the surface free energy (SFE) [41]. 

The profilometry analysis was made with a Form Talysurf^®^ I–Series PRO Range Taylor Hobson Ametek (Warrenville, IL, USA) device and measures *roughness* according to ISO 21920. It uses Metrology 4.0 Software (Taylor Hobson Ametek, IL, USA) and has a transducer with a standard probe. 

Based on five observations, we determine the Ra (arithmetic average deviation from the mean line) and Rq (root mean square average of the profile heights over the evaluation length) parameters. 

## 3. Results and Discussion

### 3.1. Microstructural Characterization of the Alloys

**Figure 2:**
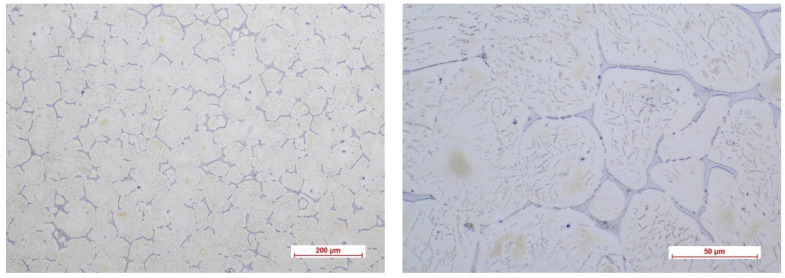
The optical micrographs corresponding to the Mg3Nd_A alloy (10× and 50×).

**Figure 3:**
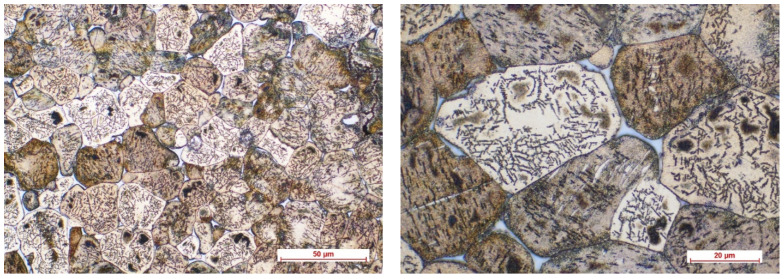
The optical micrographs corresponding to the Mg3Nd_A alloy (50× and 100×) after metallographic etching.

**Figure 4:**
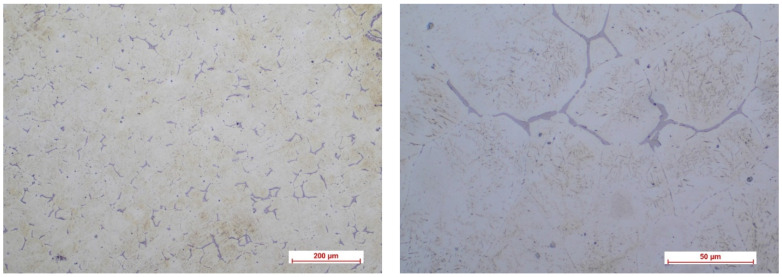
The optical micrographs corresponding to the Mg3Nd_B alloy (10× and 50×).

**Figure 5:**
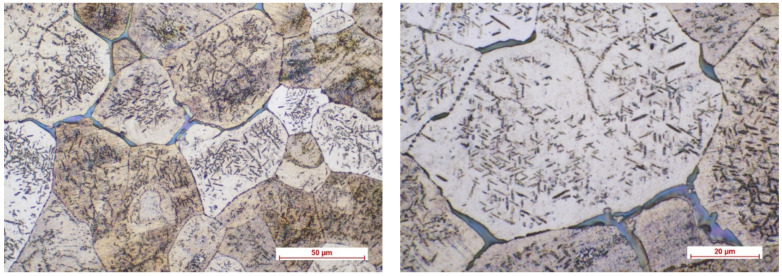
The optical micrographs corresponding to the Mg3Nd_B alloy (50× and 100×) after metallographic etching.

Figure 2, Figure 3, Figure 4 and Figure 5 show the optical micrographs corresponding to Mg3Nd_A and Mg3Nd_B alloys before and after metallographic etching at different magnifications.

The alloys’ microstructure is formed by uniform polyhedral grains of α-Mg, in which secondary phases are precipitated, with different morphologies, from acicular to globular, uniformly distributed in the α-Mg grains. The micrographs also highlight a distinct phase at the grain boundary, most likely a multicomponent intermetallic compound. In the Mg3Nd_A alloy, compared to the Mg3Nd_B alloy, the grains are smaller and more uniform in size, the degree of intragranular precipitation is higher, and acicular (icosahedral) compounds are the majority, tending to group “in packages” which will assure better mechanical properties for these alloys. The effect of the presence of a larger amount of Y in the chemical composition of the Mg3Nd_A alloy, as well as of Nd, materializes through the uniformity of the grain size, simultaneously with its reduction. Also, the proportion of the phase at the grain boundaries is visibly reduced in the Mg3Nd_B alloy, becoming insular, which will increase the alloy’s ductility.

Other studies found similar results regarding the Y and Nd effects on the alloy microstructure. Xu et al. [42] prepared through the extrusion technique the Mg-Zn-Y-Zr alloy. They varied the yttrium composition between 0 and 3.5 wt.%, proving that Y addition has a grain refinement effect and increased mechanical strength by forming the I-phase (Mg_3_YZn_6_) in the Mg matrix. At a concentration higher than 1.72 wt.%, the mechanical strength decreased due to the appearance of the W-phase (Mg_3_Y_2_Zn_3_). It was concluded that the best mechanical properties were achieved for a Y proportion between 1.17 and 1.72 wt.%. Wang et al. [43] investigated the influence of Nd content on the microstructure of Mg4.5Zn1YxNd0.5Zr (x = 0, 1 wt.%, 2 wt.%, 3 wt.%). Optical microscopy revealed that in the case of 0 wt.% alloy, the main phases are α-Mg, Mg_3_Y_2_Zn_3_ (W), Zn-Zr, and Mg_3_YZn_6_ (I). When Nd was added, the I-phase was not observed anymore, and Mg_3_Y_2_Zn_3_ changed to Mg_3_(Nd, Y)_2_Zn_3_. It was observed that at 3 wt.% Nd a ternary phase of Mg-Zn-Nd occurred, concomitantly to an increase in intermetallic phases and a reduction in secondary dendritic arm spacing. Zhou et al. [44] performed an investigation regarding the effect of Y and Nd addition to the Mg6Zn0.6Zr (ZK60) alloy. They observed two new phases, Mg_3_Zn_6_Y (I-phase) and Mg_41_Nd_5_, grain refinement, and improved mechanical properties. Zengin and Turen [45] analyzed the effect of Y addition on the microstructure of Mg6Zn0.5Zr1NdxY (x = 0, 0.5, 1, 2). They noticed that the as-cast microstructure obtained for x = 0 contained α-Mg and also Mg-Zn-Nd and Mg-Zn phases. With the Y addition appeared two ternary phases, W-phase (Mg_3_Zn_3_Y_2_) and I-phase (Mg_3_Zn_6_Y), associated with the grain refinement process. 

**Figure 6:**
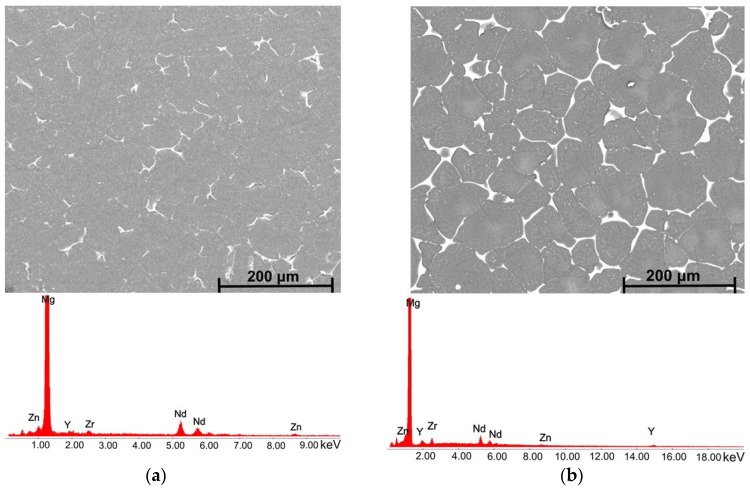
SEM images and EDS spectra for (**a**) Mg3Nd_B alloy; (**b**) Mg3Nd_A alloy.

**Figure 7:**
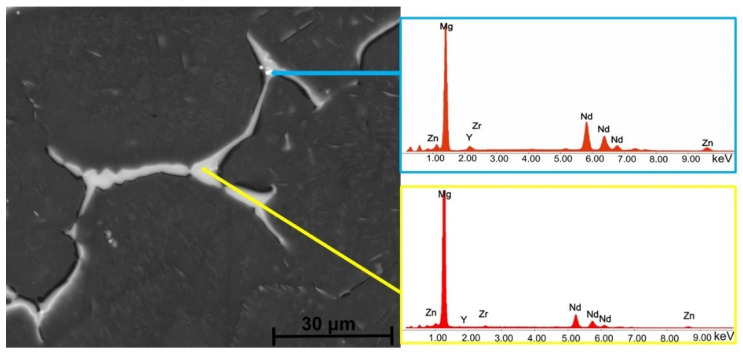
SEM image and associated EDS spectra for Mg3Nd_A alloy.

**Figure 8:**
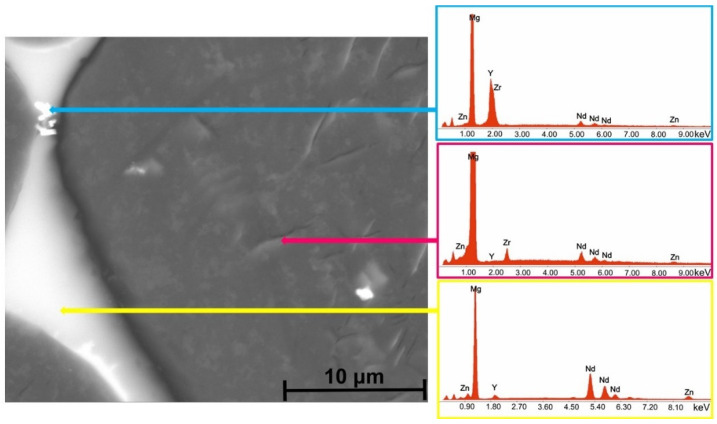
SEM image and associated EDS spectra for Mg3Nd_B alloy.

**Figure 9:**
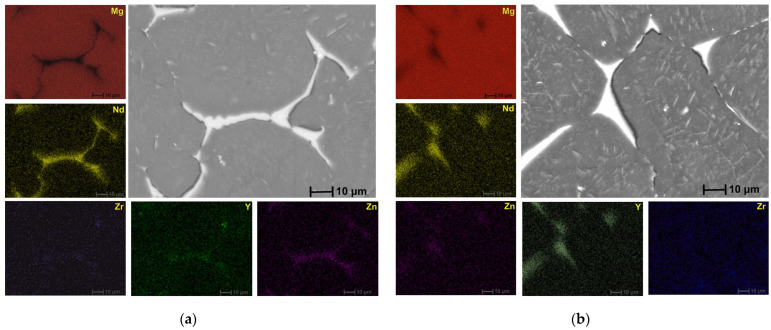
EDS elemental mapping results on (**a**) Mg3Nd_A alloy; (**b**) Mg3Nd_B alloy.

Figure 6, Figure 7, Figure 8 and Figure 9 reveal the SEM images and EDS analysis of the Mg3Nd_A and Mg3Nd_B alloys at different magnifications. Results sustain the optical investigation, highlighting the morphology and distribution of secondary phases. 

According to the EDS results for the Mg3Nd_B alloy, the phase identified at the grain boundary proved to be rich in Mg, Zn, and Nd. Increasing the amount of Y in the alloy composition (Mg3Nd_A alloy), the formation of a grain boundary rich in Mg, Zn, Nd, and Y is observed. Elkaimey et al. [46] and Zengin et al. [45] proposed that the phase be identified as Mg_3_(Nd, Y)_2_Zn_3_. Moreover, the granular compounds present in the alloy structure contain rare earths (Y and Nd), while the acicular compounds are composed of Zr and Zn. Li et al. [47] added Nd and Y to an as-cast Mg5Zn0.6 Zr alloy, and α-Mg phase and compounds such as Mg_4_Zn_7_ and Zn_2_Zr_3_ were observed. Also, a refined microstructure with an increase in the secondary phase was put in evidence. 

**Figure 10:**
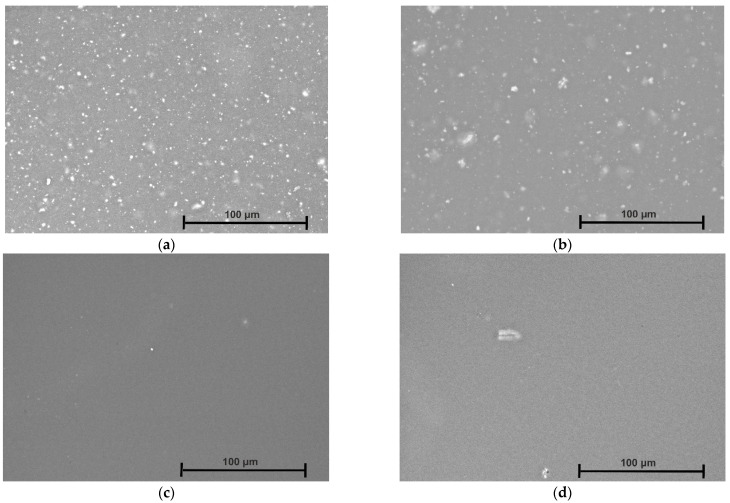
SEM images of the coated Mg3Nd alloys: (**a**) Mg3Nd_A_CAHAp; (**b**) Mg3Nd_A_CAMg; (**c**) Mg3Nd_B_CAHAp; (**d**) Mg3Nd_B_CAMg.

**Figure 11:**
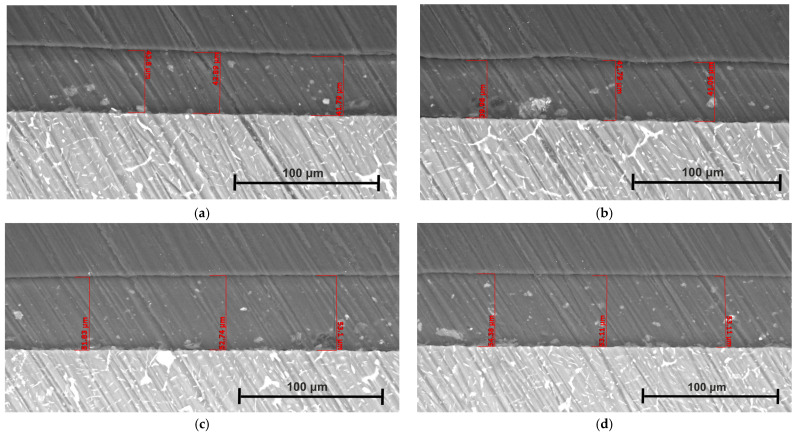
Coating thickness deposited on the Mg3Nd alloy surfaces: (**a**) Mg3Nd_A_CAHAp; (**b**) Mg3Nd_A_CAMg; (**c**) Mg3Nd_B_CAHAp; (**d**) Mg3Nd_B_CAMg.

The surface morphology of the coated Mg3Nd alloys and the coating thickness are presented in Figure 10 and Figure 11.

From the SEM images, we can observe that in both cases, the polymeric composite coatings with Mg or HAp particles exhibit a uniform aspect that evidences a homogenous distribution of the particles in the polymeric matrix. Pores with different distributions and average sizes were formed due to the N,N′-dimethylformamide evaporation process, whose particles diffused outside the sample surface. The main conclusion that can be extracted is that the developed coatings are very well prepared, and in the case of composite coating with Mg particles, a much smoother surface is evidenced. The cross-section images underline the abovementioned findings and show that both coatings are homogenously placed on the Mg3Nd alloy surface. We determined, for the coating thickness in the case of Mg3Nd_A alloy, values of 42.8 μm (Mg3Nd_A_CAHAp) and 52.5 μm (Mg3Nd_A_CAMg). 

For the other alloy (Mg3Nd_B), the coating thickness was about 40.9 μm (Mg3Nd_B_CAHAp) and 53.5 μm (Mg3Nd_B_CAMg). Due to the different particle natures and interaction forces between CA and the reinforcing agent, the coating thickness exhibits lower values in the case of composite coating with HAp particles.

### 3.2. Corrosion Behavior of the Experimental Samples

#### 3.2.1. Immersion Test

**Figure 12:**
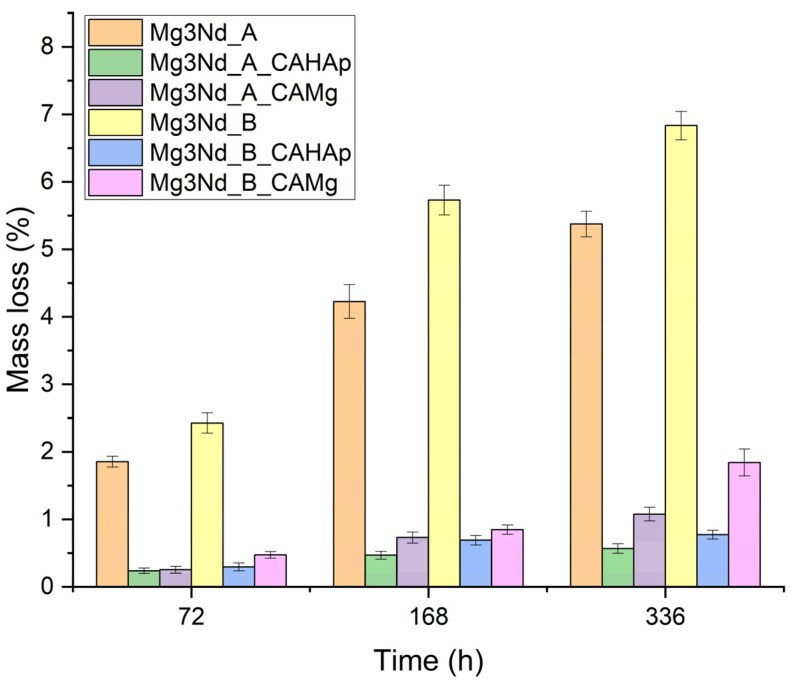
Mass loss evolution of the uncoated and coated Mg3Nd alloys in SBF solution.

The mass loss values obtained by immersing the experimental samples in the SBF solution after 72, 168, and 336 h are presented in Figure 12.

The specific reactions of the magnesium and its alloy corrosion process in aqueous media produce magnesium hydroxide and hydrogen [48] according to the following reactions:

(5) $$Anodic\;reaction:Mg\;↔\;{Mg}^{2+}+{2e}^{-}$$

(6) $$Cathodic\;reaction:{2H}_{2}O+{2e}^{-}↔H_{2}+{2HO}^{-}$$

(7) $$Overall\;reaction:Mg+{2H}_{2}O\;↔\;{Mg}^{2+}+H_{2}+{2HO}^{-}$$

(8) $$Product\;formation:Mg+{2HO}^{-}\;↔\;{Mg(OH)}_{2}$$

The anodic reaction takes place inside the α-Mg matrix, which, due to a very negative electrode potential, is prone to be galvanically attacked. When the concentration of chlorine ions (Cl^−^) from the composition of the corrosion environment exceeds 30 mmol/L, the magnesium hydroxide layer will react with Cl^−^ to form magnesium chloride (MgCl_2_, highly soluble) and hydroxyl ions. The concentration of chlorine ions in the physiological medium is around 150 mmol/L, which is much higher than the concentration that the magnesium hydroxide layer can support. As a result, Mg alloys will suffer severe corrosion and rapid degradation in vivo. When the Mg(OH)_2_ layer reacts with Cl^−^ ions, it releases hydroxide ions (OH)^−^ leading to a local increase in pH.

(9) $$Mg{\left(OH\right)}_{2}+{2Cl}^{-}↔{MgCl}_{2}+{2HO}^{-}$$

The lowest values were recorded for Mg-based alloys coated with composite coatings based on cellulose acetate with HAp particles (0.56% for Mg3Nd_A_CAHAp at 336 h and 0.77% for Mg3Nd_B_CAHAp at 336 h). For the uncoated alloys, the mass loss at 336 h is 5.37% for the Mg3Nd_A alloy and 6.83% for the Mg3Nd_B alloy. The results highlight yttrium’s beneficial effect on the alloys’ corrosion behavior. A higher proportion of Y (2.10% in the Mg3Nd_A alloy compared to 0.21% in the Mg3Nd_B alloy) generates a lower mass loss and, therefore, a higher corrosion resistance due to the alloy microstructure’s refinement. For the uncoated alloys (Mg3Nd_A and Mg3Nd_B), a higher mass loss is observed during the first 168 h of immersion. After this time, the corrosion process is reduced in intensity due to the increase in pH to a value at which the Mg(OH)_2_ layer is stable on the surface of the magnesium alloys. Also, the results reveal that the composite coatings made on the Mg3Nd alloy surface provide protection against the test environment. The results obtained for the Mg3Nd_A_CAHAp and Mg3Nd_B_CAHAp samples are 10 and 9 times lower than the uncoated alloys, respectively, and for the Mg3Nd_A_CAMg and Mg3Nd_B_CAMg samples, 5 and 4 times lower than the uncoated alloys. 

The results showed that, in the case of both Mg3Nd alloys, the composite coating based on CA with HAp particles achieves the best protection against the test environment.

#### 3.2.2. Electrochemical Tests

**Figure 13:**
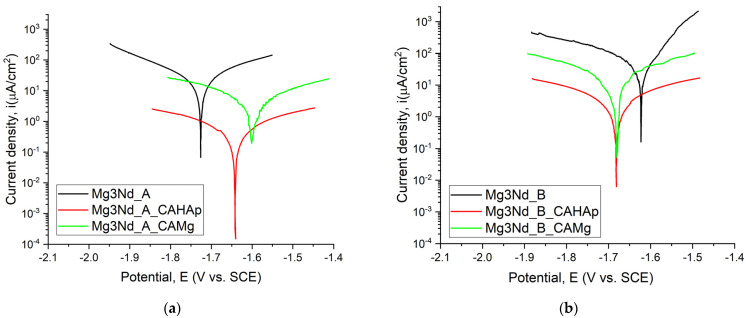
Tafel variations of the uncoated and coated samples: (**a**) Mg3Nd_A; (**b**) Mg3Nd_B.

The Tafel curves for coated and uncoated samples are presented in Figure 13.

The electrochemical measurements for the uncoated and coated alloys are presented in Table 3.

The corrosion resistance of the uncoated and coated Mg3Nd samples was analyzed based on different evaluation criteria. It is well known that a more electropositive value of the open circuit potential (*E_oc_*) is directly linked to a nobler character from an electrochemical point of view and a much better corrosion behavior in SBF. By analyzing the values presented in Table 3, it can be noticed that all the coated samples exhibited better corrosion resistance than uncoated alloys. The much more electropositive values of *E_oc_* were obtained for Mg3Nd_A_CAMg (−1.610 V) and Mg3Nd_A_CAHAp (−1.647 V) at a difference of only 0.37 V. Also, the Mg3Nd_B_CAMg (−1.696 V) and Mg3Nd_B_CAHAp (−1.683 V) were characterized by good corrosion behavior. If we consider the corrosion potential criteria (*E_corr_*), the more electropositive values are obtained for the coated samples, which are ranked as follows: Mg3Nd_A_CAMg (−1.605 V), Mg3Nd_A_CAHAp (−1.641 V), Mg3Nd_B_CAMg (−1.678 V), and Mg3Nd_B_CAHAp (−1.681 V).

Regarding the corrosion current density, the smallest value shows a higher corrosion resistance. In our case, the lowest value of *i_corr_* was achieved for the samples with a composite coating based on CA and HAp particles (i.e., Mg3Nd_A_CAHAp–4.497 μA/cm^2^, Mg3Nd_B_CAHAp–14.971 μA/cm^2^). Also, a good corrosion behavior was noticed for the samples coated with composite coatings based on CA and Mg particles, but the values of the corrosion current density were higher than those previously mentioned (Mg3Nd_A_CAMg–16.684 μA/cm^2^, Mg3Nd_B_CAMg–20.607 μA/cm^2^). It can be concluded that both composite coatings offered very high protection against corrosion. From the polarization resistance (*R_p_*) point of view, a higher value of this parameter indicates an increased corrosion resistance. The electrochemical tests made in SBF showed that Mg3Nd_A_CAHAp (87.210 kΩcm^2^) and Mg3Nd_B_CAHAp (12.847 kΩcm^2^) exhibited the best corrosion behavior. The composite coating based on CA and Mg particles offered medium protection against corrosion (Mg3Nd_A_CAMg–8.469 kΩcm^2^, Mg3Nd_B_CAMg–2.819 kΩcm^2^) in comparison with the uncoated samples that exhibited the lowest value of the polarization resistance (Mg3Nd_A–1.530 kΩcm^2^, Mg3Nd_B–0.535 kΩcm^2^). The corrosion rate can be computed only in the case of uncoated samples, and it can be seen that the Mg3Nd_A had a lower value (0.885 mm/year) than Mg3Nd_B (1.266 mm/year). The protective efficiency must have a higher value to indicate the protective effect of the coating against the corrosion phenomenon. This parameter can be computed only in the case of coated samples. It can be noticed that the best protective efficiency is offered by composite coatings based on CA with HAp or Mg in the case of the Mg3Nd_A alloy. The same order is observed for the Mg3Nd_B material, but the protective efficiency had lower values.

Gaon et al. [49] investigated the effect of the local microstructure of Mg-Zr-Nd-Y-based alloys on their corrosion behavior compared with other magnesium-based alloys. A skin effect was noticed for the higher Y content alloy during the corrosion process due to the higher content of Mg3Nd phase. The authors found a correlation between the grain structure and corrosion rate and stated that a coarser grain determined an increased corrosion rate. They found that the mass loss (*P_w_*) for the Mg-Zr-Nd-Y-based alloy with Y higher content was about 1.46 ± 0.04 mm/year and a gas evolution (*P_H_*) of about 1.29 ± 0.06 mm/year, while for the Mg-Zr-Nd-Y-based alloy with a lower Y content, *P_w_* was equal to 6.21 ± 0.05 mm/year and pH was 7.73 ± 0.05. It can be observed that an increased Y content generated a higher corrosion resistance.

Zemkova et al. [50] analyzed the effect of rare earth addition on the corrosion behavior of Mg3Nd (N3), Mg-3Y (W3), and Mg-4Y-3Nd (WN43). They noticed that Mg-Y secondary phase particles had a detrimental effect on the corrosion process by reducing the value of the corrosion resistance [51,52], since for the case in which Y is dissolved in the magnesium matrix, the corrosion resistance increased [53]. Regarding the WN43 alloy, the second phase Mg- (Nd, Y) did not generate a severe galvanic corrosion effect because the potential difference between the secondary phase and α-Mg matrix is only about 25 mV [54]. 

As a final observation from the protective effect of the coating, the best protective efficiency for both the Mg3Nd alloys is offered by the composite coating based on CA with HAp particles. The composite coating based on CA with Mg particles was also efficient but less than the coating mentioned before. The two developed films determined increased corrosion resistance and have great potential to be used as coatings for Mg-based implants.

### 3.3. Surface Properties of the Experimental Samples

#### 3.3.1. Wettability

**Figure 14:**
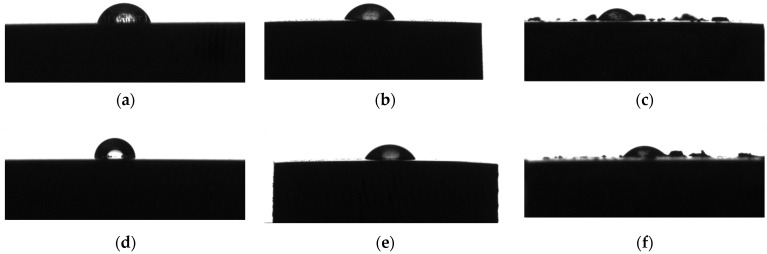
Examples of droplet shape and average values obtained for the experimental samples. For Mg3Nd_A: (**a**) control sample (70.37 ± 2.52°); (**b**) Mg3Nd_A_CAHAp (64.23 ± 1.19°); (**c**) Mg3Nd_A_CAMg (53.89 ± 1.98°); for Mg3Nd_B; (**d**) control sample (85.91 ± 2.02°); (**e**) Mg3Nd_B_CAHAp (57.21 ± 1.95); (**f**) Mg3Nd_B_CAMg (52.79 ± 2.62). Wetting agent–water.

The wettability investigation is important to establish if the material surface represents a proper medium for an adequate biological response. It is well known that in the case of high hydrophilicity, cell proliferation and adhesion increase, and a better osteointegration of the implant occurs. Higher than 90° contact angles characterize a hydrophobic surface, which sometimes is not favorable to protein and molecule absorption from the biological fluids. Figure 14 presents examples of droplets for the Mg3Nd_A and Mg3Nd_B alloys in the case of water as a wetting agent for control (uncoated sample) and coated samples. It can be observed that in the case of Mg3Nd_A and Mg3Nd_B alloys, the composite coatings based on CA reinforced with HAp or Mg particles transform the alloy surface into a more hydrophilic one, with the lowest value of contact angle (52.79°) obtained for the Mg3Nd_B_CAMg.

The HAp particles included in the composite coating determine the hydrophilic behavior of the surface with a contact angle of about 57.21° (Mg3Nd_B_CAHAp) and 64.23° (Mg3Nd_A_CAHAp). It can be concluded that the coating procedure in the case of both the Mg-REs alloys is directly linked to hydrophilization of the surface with a much more predominant effect in the case of Mg3Nd_B alloy.

**Figure 15:**
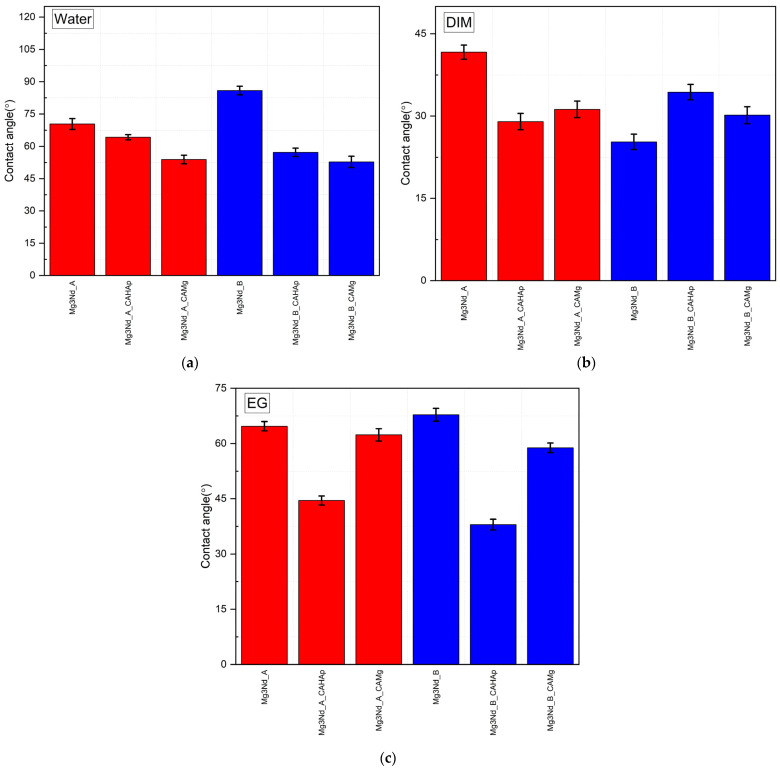
Contact angles (average values and standard deviations) for the Mg-REs alloys for three wetting agents: (**a**) water; (**b**) diiodomethane; (**c**) ethylene glycol.

Figure 15 shows the graphs for the tested samples obtained in the case of the three wetting agents. For the diiodomethane liquid, the Mg3Nd_A_CAHAp and Mg3Nd_B exhibited the most hydrophilic characters, with a contact angle of about 29 ± 1.49° and 25.3 ± 1.4°. In the case of the ethylene glycol wetting agent, the samples with a composite coating based on CA with HAp particles have the lowest contact angle value (i.e., Mg3Nd_A_CAHAp–44.53°, Mg3Nd_B_CAHAp–37.99°).

**Figure 16:**
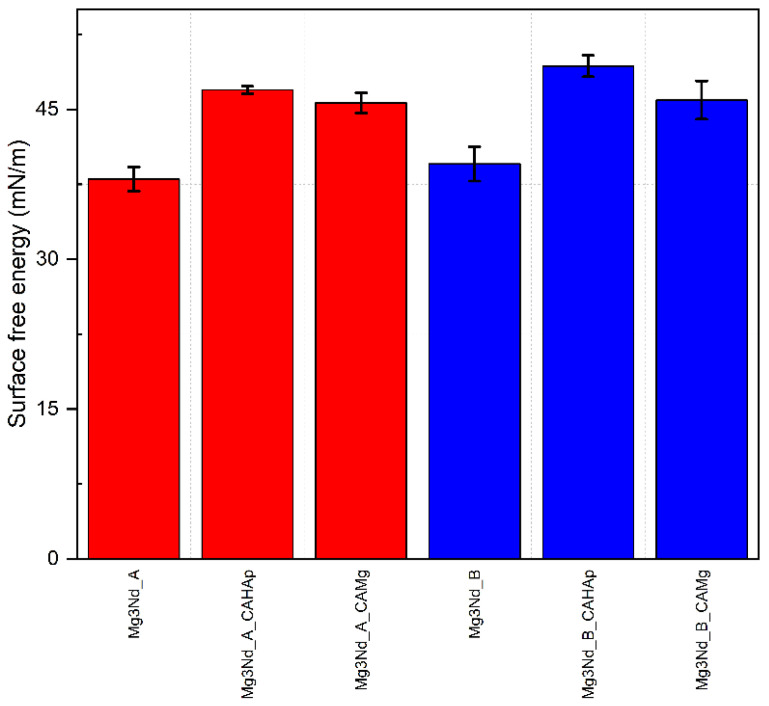
Surface free energy (SFE) results of the investigated samples computed with OWKR method.

Based on the fact that ethylene glycol (EG) and water (W) are considered polar liquids, while diiodomethane (DIM) is a nonpolar/dispersive liquid, and their surface energy components are known as reported in [41], in Figure 16 the alloy surface free energy (SFE) is shown after the OWKR method was applied. The surface wettability can be directly correlated with SFE. Higher energy values are usually associated with lower values of contact angles and are characteristic of a surface with increased cell proliferation properties and absorption of biological substances [55,56,57,58,59]. The cell behavior could be predicted more accurately just after a complex surface characterization, including chemical composition, topography, roughness, and wettability.

In our case, the samples coated with composite coatings based on CA with HAp particles exhibited the highest value of SFE, since the lowest values characterized the uncoated samples.

**Figure 17:**
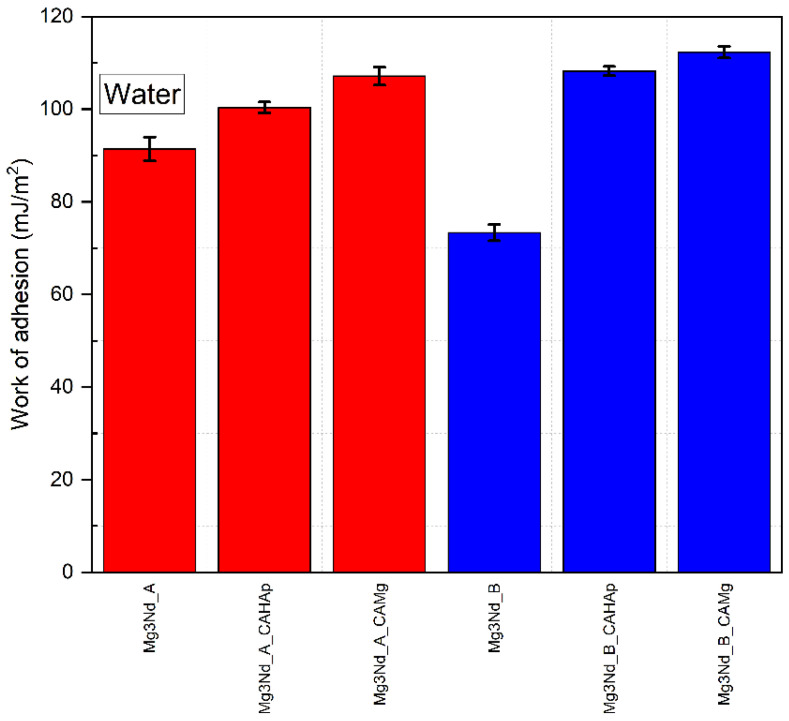
Work of adhesion in the case of water as the wetting agent for the tested samples.

The work of adhesion represents the reversible thermodynamic work, which is necessary to separate the interface in an equilibrium state between two phases if an infinite distance is considered. The obtained values are presented in Figure 17 for water as a wetting agent. An increased interfacial attraction determines a high work of adhesion value. In the case of Mg3Nd_A alloy, a value of 91.4 ± 2.58 mJ/m^2^ was obtained. After the composite coatings based on CA with HAp or Mg particles were applied, the work of adhesion increased to 100.36 ± 1.18 mJ/m^2^ and 107.13 mJ/m^2^, respectively. For the Mg3Nd_B alloy, the work of adhesion increased from 73.31 ± 1.77 mJ/m^2^ to 108.22 ± 0.98 mJ/m^2^ (Mg3Nd_B_CAHAp sample) and 112.28 ± 1.24 mJ/m^2^ (Mg3Nd_B_CAMg sample).

From the contact angle analyses, it can be concluded that the samples with a composite coating based on CA with HAp are the most beneficial to cell adhesion and proliferation and adequate for increased osteogenesis. Our analysis can be considered in good accordance with the literature. In the case of the Mg-Zn-Y-Nd alloy used as potential material for biodegradable stent manufacture, Wang et al. [60] obtained a contact angle of about 60°. Indira et al. [61] studied the wettability properties of laser surface-modified rare earth Mg alloys. They used the commercial Mg4Y3Nd0.5Zr (WE43) alloy in their studies and measured a contact angle for the control sample of 81° and 61° for deionized water and SBF as wetting agents. Their main conclusion was that the laser surface treatment modified the surface character by transforming it into a more hydrophilic one (i.e., a contact angle of 43° was obtained for deionized water). The small differences between our results and those found in the literature can be attributed to the different materials’ chemical compositions and concentrations of rare earths. 

The results regarding the contact angle measurements were compared using one-way ANOVA assuming equal variances with a confidence level of 95% associated with Tukey and Fisher’s simultaneous tests of the means. It can be concluded, at α = 0.05, that the means are significantly different—the surface modification is an influence factor for wetting behavior. The Tukey and Fisher tests yielded that through groups, some means can be considered equal, an aspect that was expected given the same nature of the coating. The differences between the means for the coatings can be attributed to the roughness and dispersion of the HAp and Mg particles, easily observed in the averages of the surface free energy: a significant increase post-coating and small differences between coated samples. The main contribution in surface-free energy can be attributed to the matrix of the coating, while the reinforcing phase shows a milder one.

#### 3.3.2. Roughness

**Figure 18:**
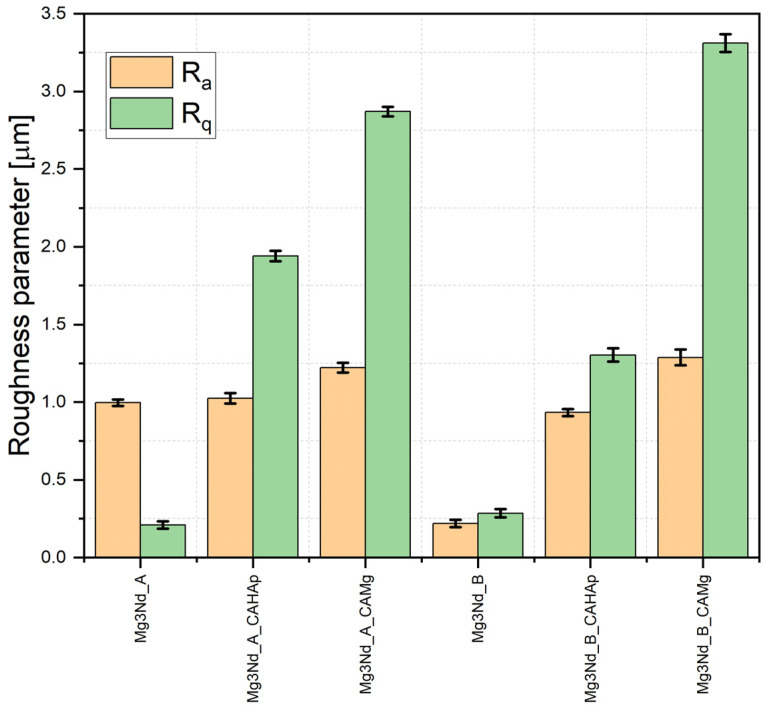
*R_a_* and *R_q_* parameters for surface roughness of the investigated alloys.

The surface roughness is an important parameter that should be taken into consideration when implant integration in the human body is necessary. The literature revealed that roughness had a direct influence on the material degradation rate [62,63,64], cell adhesion and proliferation [65,66], and osteointegration [67,68]. Some research showed that a rough surface had a beneficial effect in the reduction in the degradation rate [69,70,71,72], since other investigations sustained the contrary [66,73]. The surface topography was investigated based on profilometry analysis. Our results revealed that the uncoated samples had the lowest values of the *R_a_* and *R_q_* parameters (e.g., Mg3Nd_A: *R_a_* = 0.996 ± 0.021 μm, *R_q_* = 0.208 ± 0.023 μm; Mg3Nd_B: *R_a_* = 0.218 ± 0.022 μm, *R_q_* = 0.284 ± 0.026 μm). In the case of the first alloy (Mg3Nd_A), the composite coating based on CA with Mg particles generated a higher increase in the *R_q_* parameter (*R_q_* = 2.87 ± 0.030 μm) in comparison with the sample with composite coatings based on CA and HAp particles (*R_q_* = 1.94 ± 0.033 μm), since regarding the *R_a_* parameter, similar values of about 1.222 ± 0.032 μm (Mg3Nd_A_CAMg) and 1.024 ± 0.034 μm (Mg3Nd_A_CAHAp) were achieved. For the second investigated alloy (Mg3Nd_B), the highest values of the roughness parameters were obtained in the case of composite coating based on CA with Mg particles (*R_a_* = 1.288 ± 0.050 μm, *R_q_* = 3.310 ± 0.057 μm), followed by composite coating based on CA with HAp particles (*R_a_* = 0.933 ± 0.022 μm, *R_q_* = 1.304 ± 0.042 μm) (Figure 18).

A correlation between surface roughness and wettability was given in the literature by the Wenzel model [74]. This theory states that an increase in the roughness will determine a decrease in the contact angle in the case of hydrophilic surfaces. This observation is valid in our case for both alloys and water as a wetting agent, correlating to an increase in the roughness surface due to coating procedures with a more hydrophilic surface (Figure 15a). A similar one-way ANOVA was performed on the roughness results, yielding that the mean values are different; the Tukey and Fisher tests suggest that the only means that can be considered equal for Ra are those of Mg3Nd_A and Mg3Nd_A_CAHAp.

## 4. Conclusions

The present study investigated the effect of CA-based composite coatings reinforced with hydroxyapatite or magnesium particles applied on the two Mg3Nd alloys on their surface properties and corrosion behavior. The SEM images revealed the presence of homogenous coating layers in both the coatings, which ensure a high protective behavior against the corrosion process. In addition, the CA-based composite coatings determined a decrease in contact angle and an increase in roughness values, a fact that evidences the existence of an environment that is favorable for the promotion of cell growth, proliferation, and adhesion.

The in vitro research highlighted that the composite coating based on CA with HAp particles exhibited the best functional properties for both of the investigated Mg3Nd alloys. The composite coating based on CA with Mg particles was also efficient but in a reduced amount. Considering the results presented in this study, we can conclude that the proposed CA-based composite coatings have great potential to be used in coated Mg alloy implant manufacture.

## Figures and Tables

**Table 1:** Synthetic polymer coatings applied on Mg-based alloys.

Coating	Mg-Based Substrate	Remarks	Ref
PLA	Mg9Al1Zn	The authors investigated the degradation of samples in simulated body fluid (SBF) based on electrochemical impedance spectroscopy (EIS). It was concluded that a thickness of 5 μm for the polymeric coatings determines a corrosion resistance increase of two times compared to the bare Mg alloy. Thicker PLA layers were linked to poor adhesion.	Alabbasi et al. [33]
PLA/micro-arc oxidation (MAO)	Mg3Al1Zn	After the MAO process was applied, a 5 μm porous MgO layer was obtained. Then, the samples were immersed in PLA, and a coating with 9 μm thickness was formed. Following the electrochemical corrosion analysis in the case of coated samples, the corrosion potential was about −1.51 V, and the corrosion current density had a very low value, equal to 1.83 μA/cm^2^.	Shi et al. [34]
PCL (3% (*w*/*v*)) and PCL (6% (*w*/*v*))	Mg-based scaffolds	It was noticed that the uncoated scaffolds fully degraded after 72 h, and the coated samples degraded in a proportion of 36% and 23%.	Yazdimamaghani et al. [35]
PLGA	Mg6Zn	PLGA was dissolved in chloroform with 2 wt.% and 4 wt.% concentrations. The corrosion analysis revealed a very low value for the corrosion current density in the coated samples compared to the control one.	Li et al. [36]
(MAO) + PLGA	Mg4Zn0.6Zr0.4Sr	Compared with the uncoated sample, the (MAO) + PLGA coatings increased the corrosion resistance of the alloy by three orders of magnitude, and the stress corrosion cracking susceptibility measurements were reduced by 75% of the ultimate tensile strength.	Chen et al. [37]
Composite coatings based on CA, CA-HAp, CA-Mg, and CA-HAp-Mg	-	They noticed that the thermogravimetric analysis proved the coating stability up to 200 °C, highlighting a mass loss with a maximum value of 9% at a maximum temperature of 250 °C. HAp crystals were put in evidence through scanning electron microscopy investigations. The CA-Mg and CA-HAp composite coatings were characterized by a viability of 80% for the MG-63 cell line.	Streza et al. [38]

**Table 2:** Elemental composition of the Mg3Nd alloys.

Alloys	Composition (wt.%)				
	Zn	Zr	Y	Nd	Mg
Mg3Nd_A	0.3	0.6	2.10	3.2	Bal
Mg3Nd_B	0.3	0.4	0.21	3.1	Bal

**Table 3:** The electrochemical parameters obtained on the coated and uncoated Mg3Nd alloys.

Sample ID	E_oc_ (V)	E_corr_ (V)	i_corr_ (µA/cm^2^)	β_c_ (mV)	β_a_ (mV)	CR (mm/Year)	P_e_ (%)	R_p_ (kΩcm^2^)
Mg3Nd_A	−1.765	−1.720	38.918	266.60	280.011	0.885	-	1.530
Mg3Nd_A_CAHAp	−1.647	−1.641	4.497	2637	1371	-	99.63	87.210
Mg3Nd_A_CAMg	−1.610	−1.605	16.684	625.154	676.912	-	98.66	8.469
Mg3Nd_B	−1.698	−1.629	55.689	268.870	87.419	1.266	-	0.535
Mg3Nd_B_CAHAp	−1.683	−1.681	14.971	1008	788.346	-	98.54	12.847
Mg3Nd_B_CAMg	−1.696	−1.678	20.607	297.275	242.764	-	97.99	2.819

## Data Availability

Data are contained within the article.
